# Targeted expression of tumor necrosis factor-related apoptosis-inducing ligand TRAIL in skin protects mice against chemical carcinogenesis

**DOI:** 10.1186/1476-4598-10-34

**Published:** 2011-04-04

**Authors:** Valerie Kedinger, Stephanie Muller, Hinrich Gronemeyer

**Affiliations:** 1 Department of Cancer Biology, Institut Génétique de Biologie Moléculaire et Cellulaire (IGBMC), BP 10142, 67404 Illkirch-Cedex, C.U. de Strasbourg, France

## Abstract

**Background:**

Gene ablation studies have revealed that tumor necrosis factor-related apoptosis-inducing ligand (TRAIL, Apo2L, TNFSF10) plays a crucial role in tumor surveillance, as TRAIL-deficient mice exhibit an increased sensitivity to different types of tumorigenesis. In contrast, possible tumor-protective effect of increased levels of endogenous TRAIL expression *in vivo *has not been assessed yet. Such models will provide important information about the efficacy of TRAIL-based therapies and potential toxicity in specific tissues.

**Methods:**

To this aim, we engineered transgenic mice selectively expressing TRAIL in the skin and subjected these mice to a two-step chemical carcinogenesis protocol that generated benign and preneoplastic lesions. We were therefore able to study the effect of increased TRAIL expression at the early steps of skin tumorigenesis.

**Results:**

Our results showed a delay of tumor appearance in TRAIL expressing mice compared to their wild-type littermates. More importantly, the number of tumors observed in transgenic animals was significantly lower than in the control animals, and the lesions observed were mostly benign. Interestingly, Wnt/β-catenin signaling differed between tumors of wild-type and TRAIL transgenics.

**Conclusion:**

Altogether, these data reveal that, at least in this model, TRAIL is able on its own to act on pre-transformed cells, and reduce their tumorigenic potential.

## Background

Tumor necrosis factor-related apoptosis-inducing ligand (TRAIL, Apo2L, TNFSF10), a type II trans-membrane death ligand, has the unique property of inducing apoptosis in tumor cells while sparing normal ones [1]. The human protein shares 65% amino-acids identity with its murine counterpart [2]. TRAIL forms homotrimers that ligate to two types of receptors: death receptors that trigger TRAIL-induced apoptosis and decoy receptors that can antagonize apoptosis induction. In humans, two death receptors (DR4/TRAIL-R1/TNFRSF10A and DR5/TRAIL-R2/TNFRSF10B) [3,4] and two decoy receptors (DcR1/TRAIL-R3/TNFRSF10C and DcR2/TRAIL-R4/TNFRSF10D) [5] have been identified. In mice two decoy receptors (mDcR1 and mDcR2) have been characterized [6] but only one death-inducing receptor (mDR5, mTRAILR2) that shares sequence homology with both human DR4 and DR5 was found [7].

Binding of TRAIL to one of the death receptors results in receptor oligomerization and recruitment of the FAS-associated protein with death domain (FADD), which itself recruits membrane proximal caspases (caspase 8 and 10). The resulting protein complex has autocatalytic activity and is designated as the death-inducing signaling complex (DISC). The activation of this complex induces activation of a caspase cascade, which cleaves numerous proteins and ultimately leads to cell death [[8] and references therein].

Increasing evidence from various types of experimental models supports the notion that TRAIL can affect tumor onset and development. Indeed, tumor transplantation experiments with TRAIL- and TRAILR-deficient mice and the use of TRAIL-neutralizing antibodies revealed that endogenous TRAIL expressed in NK cells contributes to host immunosurveillance against primary tumors and metastases. Moreover, TRAIL exerts a potent tumoricidal activity in cancer cells in vitro and in vivo, causing negligible effects on normal cells when exogenously administered, an important feature of this cascade regarding its therapeutic potential [9-25]. Therefore, recombinant TRAIL, TRAIL "mimics" [26] and agonistic TRAIL receptor antibodies are attractive potential tools for anticancer therapy.

While loss-of-function studies have provided important information revealing accelerated tumor formation in TRAIL-deficient mice and thereby confirmed its implication in tumor defense, the corresponding *in vivo *gain-of-function analysis demonstrating protection against tumorigenesis by increasing endogenous TRAIL levels in the animal or a given tissue has not yet been done. Irrespective of the information that can be obtained with recombinant TRAIL, which corresponds only to a part of *TNFSF10*, TRAIL mimics or with agonistic TRAIL receptor antibodies, an understanding of the impact of increasing cellular TRAIL levels is an essential aspect of its biological function. Moreover, with this approach, cell and tumor-type efficacies including possible adverse effects of TRAIL therapies can be assessed, as well as the toxicity of increased levels of endogenous TRAIL *in vivo*, including effects on early development. Note that not only tumoricidal, but also increased proliferation upon exposure to recombinant TRAIL has been reported in some cases of human cancer [27] and inflammation models [28]. Finally, only *in vivo *models in which TRAIL is overexpressed in selected tissues will reveal at which stage of tumor development TRAIL overexpression becomes tumor-protective or tumoricidal, and which cell types (premalignant or malignant cells, tumor proximal normal cells, cells of the immune system) contribute. To this aim we have initiated a study in which the effect of TRAIL overexpression in the skin on chemically induced epithelial tumorigenesis was assessed.

For this we took advantage of the well-established 7,12-dimethyl-benz-anthracene/12-*0*-tetradecanoylphorbol-13-acetate (DMBA/TPA) two-stage skin carcinogenesis model. This tumor induction protocol recapitulates comprehensively the multistage nature of human epithelial cancer development [29], as it first gives rise to benign neoplastic lesions (papillomas) from which a small percentage progress into malignant squamous cell carcinomas (SCCs). In most strains of mice however, progression of benign papillomas into SCCs is a rare and late event. Ultimately, a small fraction of these tumors can metastasize to distant organs. To study the protective effect of increased endogenous TRAIL levels we engineered mice to specifically overexpress TRAIL in the skin and subjected these mice to the two-step chemical carcinogenesis protocol.

## Methods

### Generation of transgenic mice

To selectively induce expression of TRAIL in the skin, a construct was generated in which the mouse TRAIL cDNA was placed under the control of the human keratin 14 (K14) promoter. To allow a more efficient expression of the transgene, an intron of the β-globin gene, flanked on both sides by small parts of the exons 1 and 2, respectively, was introduced between the K14 promoter and the mTRAIL cDNA. The SV40 3'UTR was added at the end of the construct. This construct was microinjected into the male pronucleus of a fertilized mouse oocyte, which was than transplanted into a pseudo-pregnant recipient female.

### Carcinogenesis

The mice were housed in a temperature-controlled room with 12 h cycles of light and dark. 8 to 10 weeks old wild type and TRAIL transgenic mice were shaved to synchronize the hair cycle and treated the day after with a single dose of 50 μg DMBA (7,12-dimethyl-benzanthracene) (Sigma, St Louis, MO, USA) in 100 μl acetone. One week after DMBA application, 5 μg TPA (12-O-tetradecanoyl-phorbol-13 acetate) (Sigma) in 200 μl acetone was applied topically twice a week for 35 weeks. Control mice received acetone only. The number of tumors per mouse was weekly recorded and their size was measured with a Vernier calliper.

### In situ Hybridization

Skin samples from wild-type and TRAIL transgenic mice were collected, fixed overnight in 4% formaldehyde and embedded into paraffin. The paraffin sections were first rehydrated and primed with proteinase K, followed by dehydratation through graded ethanols. Pre-treated sections were covered with 150 μl hybridization buffer (50% formamide, 10% dextran sulfate, 1 × Denhart's solution, 10 mM Tris-HCl pH 7.5, 600 mM NaCl, 1 mM EDTA, 0.25% SDS, 0.5 mg/ml yeast tRNA) containing 100 ng/ml of DIG-labeled RNA probe, and hybridized at 55°C overnight. After hybridization, slides were washed twice with 2 × SSC, followed by 0.2 × SSC once at 45°C to remove unbound probe. The sections were then incubated with Blocking buffer (0.1 M Tris-HCl pH 7.5, 0.15 M NaCl, saturated with block reagent) for 1 h at room temperature. Alkaline phosphatase-conjugated anti-DIG antibody was diluted in blocking buffer and incubated on slides for 2 h at room temperature. Sections were then rinsed three times with detection buffer (0.1 M Tris-HCl pH 9.5, 0.1 M NaCl, 50 mM MgCl_2_), and then covered with detection buffer containing 0.18 mg/ml BCIP and 0.34 mg/ml NBT. The chromogenic reaction was carried out at 4°C for 16 h. Finally slides were washed with 1 × TE buffer, mounted with coverslips and photographed.

In order to synthesize the RNA probe, the 3' UTR of the construct introduced in mice was subcloned using the following primers:

5'ACTGACGAATTCCCCGGGGGATCCAGATCTTATT3' and

5' ACTGACCTCGAGCCAGACATGATAAGATACATTGATGAG 3'

### Semi-quantitative PCR

Skin samples from wild type and TRAIL transgenic mice were placed in 1 ml TriZol and homogenized using an TissueRuptor. RNA was extracted according to the manufacturer's protocol. A DNase treatment was performed, followed by reverse transcription. The cDNAs obtained were analyzed by PCR, using the following primers, 5' GATCCTGAGAACTTCAGG 3' and 5' CTGCTTCATCTCGTTGGTGA 3', which are specific for the transgene.

### Immunoblotting

Skin samples from wild type and TRAIL transgenic mice were disrupted in liquid nitrogen using a pestle and a mortar. The resulting powder was homogenized in RIPA buffer (1% NP 40, 0.5% Sodium-deoxycholate, 0.1% SDS in PBS) containing proetase inhibitors. Western blotting was performed according to standard protocols. Anti-TRAIL (R&D systems), anti-phospho-GSK3β (Ser9) (Cell signaling), anti-βcatenin (Upstate) and anti-Actin (Santa Cruz) antibodies were used. Secondary reagent used was horseradish peroxidase-coupled donkey anti-goat, goat anti-rabbit or goat anti-mouse antibodies (Santa Cruz).

### Histological analysis

Tumors were excised 35 weeks after DMBA/TPA application, fixed in 4% formaldehyde and embedded in paraffin. Sections of 5 μm were made using a microtome and stained with hematoxilin and eosin according to standard protocols. Pictures were taken using a Leica microscope.

### Immunofluorescence

Cryosections of lesions were rehydrated in PBS and fixed for 5 min at room temperature in 4% paraformaldehyde. After two washes in PBS 1X, tween 20 0.1%, the sections were saturated for 1 h in Normal Goat Serum (NGS) 5%, PBS 1X, tween 20 0.1% at room temperature and then incubated with primary antibodies overnight at 4°C. Keratin 1 antibody (Covance Dabco) was diluted at 1/500^e ^and α6-integrin antibody (BD Pharmingen) was diluted at 1/200^e^. Sections were than incubated for 1 h at room temperature with alexa 488 conjugated Donkey anti-Rabbit and Cy3 conjugated Donkey anti-Rat antibodies diluted at 1/400^e^, washed and counterstained with Hoechst.

### Immunohistochemistry

Paraffin sections of skin were deparaffinated in histosol and rehydrated. Antigen retrieval was performed in 10 mM sodium citrate pH 6.0 at 95°C for 10 min. Endogenous peroxidase activity was blocked by incubation in 1% H_2_O_2 _for 5 min. After 1 H of blocking, the sections were incubated over-night at 4°C with Ki67 antibody (1:1000; Tebu-Bio), in humidified chamber. The slides were then washed and incubated with biotin-linked secondary antibody, followed by the ABC elite reagent (Vector). The AEC reagent (Vector) was used as a substrate prior to counterstaining with hematoxilin and mounting.

## Results

**Figure 1:**
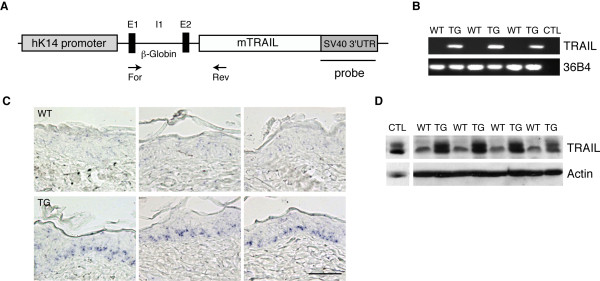
**TRAIL expression in the skin driven by the human keratin 14 promoter**. (A) Diagram of the construct introduced into ES cells. Between the mouse TRAIL cDNA and the human keratin 14 promoter, one β-globin intron and small parts of exon 1 and exon 2 were placed. The 3'UTR of SV40 was added at the end the TRAIL cDNA. The primers used for RNA expression analysis and the probe used for *in situ *hybridization are indicated on the scheme. (B) RNA was extracted from skin of wild type and transgenic animals and after reverse-transcription, semi-quantitative PCR was performed using primers specific for the transgene. 36B4 was used as a positive control. (C) RNA expression was also evaluated by *in situ *hybridization, using a probe specific for the transgene. Blue staining shows TRAIL expression in the basal cell layer of epidermis of transgenic animals. Bar, 100 μm. (D) Western blot analysis of protein extracts from wild-type and transgenic mice using an anti-TRAIL antibody and actin antibody as a loading control.

Given the potential ability of TRAIL to induce apoptosis of tumor cells, while sparing normal cells, we decided to assess whether specific TRAIL expression in the skin could prevent tumor formation following a chemical carcinogenesis. To accomplish this, we engineered mice expressing mouse TRAIL cDNA under the control of the human keratin 14 promoter (Figure 1A). It has been previously established that such constructs express the transgene specifically in the basal cell layer of the epidermis [30,31]. One mouse line was obtained in the C57BL/6 background, and tested for TRAIL expression in the skin. Both at the RNA and protein levels approximately 5-fold higher TRAIL expression was found in the skin of transgenic animals than in wild-type animals (Figure 1B and 1D). Moreover, *in situ *hybridization using a probe specific for the transgene confirmed the restricted expression of the transgene in the basal cell layer of the epidermis (Figure 1C). These mice were thus considered valid to test the effect of TRAIL overexpression on chemical carcinogenesis relative to their non-transgenic littermates.

In order to compare the tumor development in wild-type and TRAIL-expressing mice, we proceeded to a DMBA/TPA two-stage chemical carcinogenesis protocol, which consists of a single application of DMBA, followed by multiple applications of TPA for a 35 weeks period of time. While DMBA application corresponds to the initiation step of the tumor development process by inducing activating mutations of Ha-*Ras *oncogene [32,33], TPA application corresponds to the promotion step by inducing a clonal expansion of the initiated cells to form a benign tumor [34], which can eventually progress to a malignant carcinoma.

**Figure 2:**
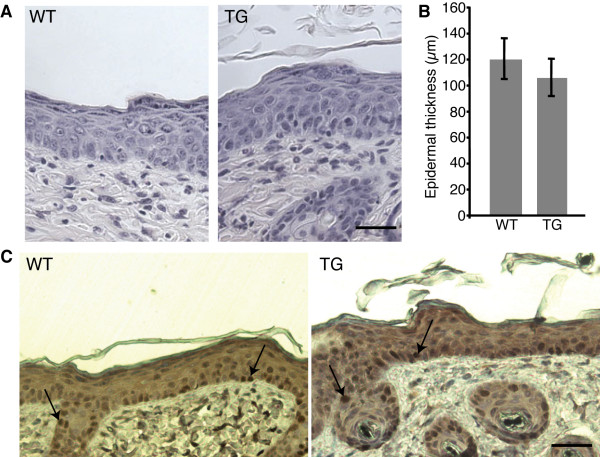
**TRAIL expression does not affect the proliferation rate induced by a short DMBA/TPA treatment**. Groups of four control and TRAIL transgenic animals were treated with DMBA/TPA for three weeks (one DMBA and four TPA applications). (A) Analysis of cell proliferation on histological skin sections with hematoxilin & eosin staining. Bar, 50 μm. (B) Quantification of epidermal thickness in micrometers, evaluated by measurements of 15 fields per animal. (C) Cell proliferation evaluated by Ki67 staining on skin sections. Bar, 50 μm.

**Figure 3:**
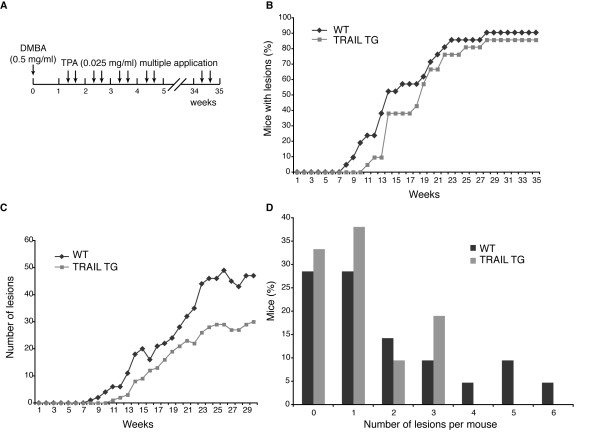
**DMBA/TPA-induced tumor formation is delayed and lower number of tumors is formed in TRAIL expressing mice compared to controls**. (A) DMBA and TPA treatment procedure. Single topical application of DMBA (0.5 mg/ml) was performed, followed by multiple applications of TPA (0.025 mg/ml), twice a week for 35 weeks. (B) Tumor incidence; percentage of mice with at least one tumor depending on time. (C) Total number of tumors in wild-type versus transgenic animals. A significant difference exists during the whole period of time of the experiment except at week 16 and week 19, as determined by Poisson distribution (D) Number of tumors per mouse; the percentage of wild-type and transgenic mice with different number of tumors was evaluated.

We first examined the skin structure of wild type and transgenic mice after a short-term treatment of DMBA/TPA, corresponding to a single application of DMBA followed by four applications of TPA. The epidermal thickening normally observed in wild-type animals after TPA treatment was not affected in TRAIL expressing mice (Figure 2A and 2B), suggesting that TRAIL expression does not impair the proliferation rate induced by TPA application. Ki67 staining of skin sections from treated wild type and transgenic mice further confirmed this notion (Figure 2C). We then started the full DMBA/TPA treatment (as illustrated in Figure 3A) on cohorts of 21 wild type and 21 transgenic mice. Whereas control animals started to develop lesions after 8 weeks of treatment, the onset of lesion formation was shortly delayed in TRAIL expressing mice, with the first appearance after 11 weeks (Figure 3B). Apart from that difference, the general tumor incidence rate was not significantly affected in TRAIL expressing mice compared to control mice, as independently of the genotype, approximately 90% of the animals developed lesions before the 30^th ^week of the treatment (Figure 3B). However, the most striking difference concerned the number of lesions observed in TRAIL expressing mice compared to control mice. Indeed, whereas the cumulative number of lesions in control animals was around 50, it was only about 30 in TRAIL transgenic mice at the end of the experiment (Figure 3C). When analyzing the number of lesions per animal, we noticed that whereas transgenic mice never presented more then three lesions, wild-type mice could display up to six lesions (Figure 3D). However, the size of the lesions was not significantly different in the wild type compared to transgenic animals (data not shown).

**Figure 4:**
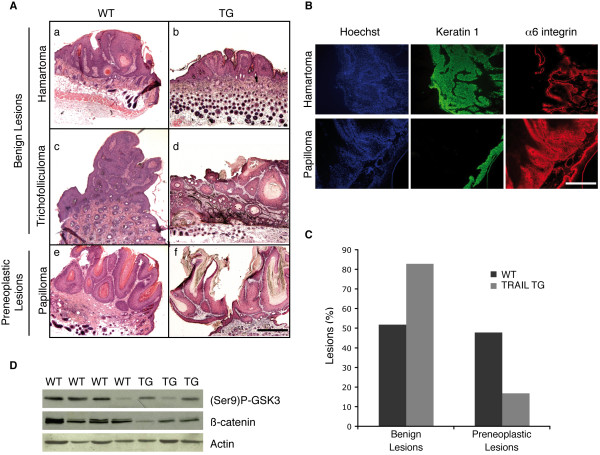
**TRAIL expressing mice develop less preneoplastic lesions compared to wild-type animals**. (A) Representative images of the histologic structure of the lesions provoqued by the treatment. DMBA/TPA treated mice develop three different types of lesions: hamartomas, trichofolliculomas and papillomas, the two first being benign whereas the last one is a preneoplastic lesion. Bar, 400 μm. (B) Representative immunofluorescence images of hamartoma and papilloma lesions stained with Keratin 1 and α6 integrin. Bar, 100 μm. (C) Percentage of benign versus preneoplastic lesions in TRAIL transgenic mice compared to wild-type mice. The difference observed between wild-type and TRAIL transgenic lesions is highly significant (P < 0.0001), as determined by Fisher's exact test. (D) Western blot analysis of wild type and transgenic tumor samples using antibodies against two transformation markers, inactivated GSK3 phosphorylated at Ser9 and β-catenin. Actin is used as a loading control.

At the end of the treatment, the lesions were excised for histological analysis. Surprisingly, none of the lesions analyzed were transformed into carcinomas. Among the benign lesions we could distinguish three different categories: hamartomas, trichofolliculomas and papillomas (Figure 4A). Whereas the two first types of lesions are benign and never transform into carcinomas, the last represents a *bona fide *preneoplastic lesion, as it can eventually evolve into a carcinoma. This classification was confirmed by immunohistochemical analysis for keratin 1 (K1) and α6-integrin expression, as it is well established that K1 expression is lost whereas α6-integrin expression is increased in lesions with high risk of transformation [35], which corresponds to our classification (Figure 4B). When quantifying the proportion of the different categories of lesions, we observed that wild type animals presented half benign and half preneoplasic lesions (Figure 4C). In contrast, less than 20% of the lesions observed in TRAIL transgenics were preneoplastic, most of them were hamartomas and trichofolliculoma (Figure 4C).

Activation of Wnt/β-catenin/Tcf signaling has been reported in non-melanocytic skin tumors induced by the two-stage carcinogenesis protocol used in the present study [36]. We therefore analyzed the expression levels of β-catenin in several tumors of both wild type and transgenic mice (Figure 4D). Most notably, β-catenin levels were lower in all the tumors extracted from TRAIL-expressing animals relative to tumors of the wild-type counterparts. Given the link between GSK3 and Wnt/β-catenin/Tcf signaling [37,38], we monitored the functional status of GSK3β in the two classes of tumors. Interestingly, the inactive form of GSK3β, which is phosphorylated at serin 9 (GSK3βpSer9), exhibited a significantly reduced expression level in transgenic lesions compared to the wild type animals (Figure 4D), suggesting that in the TRAIL overexpressing mice the development of benign lesions with active GSK3β and, possibly as a consequence, increased levels of β-catenin is disfavored. Altogether, these observations support the notion that TRAIL expression in the skin of mice partly protects them from chemical carcinogenesis already at the early steps when benign tumors form and progress.

## Discussion

In this study, we have evaluated the effect of constitutive TRAIL overexpression in a specific organ and its implication in tumor prevention following chemical carcinogenesis. Transgenic mice specifically overexpressing TRAIL in the skin were viable and were phenotypically indistinguishable from their wild type littermates. Their skin architecture was normal (data not shown); therefore, constitutive expression of TRAIL in the basal cell layer of the epidermis at the levels observed in the transgenic animals is not toxic and does not interfere with the normal development and/or homeostasis of skin.

Several reports have demonstrated that TRAIL could induce proliferation through either the ERK or the NFκB pathway [27,39,40]. In our model, we could not detect any effect on proliferation nor any activation of either ERK or NFκB when we performed either the short DMBA/TPA treatment or the complete treatment on the skin of TRAIL transgenic mice compared to wild-type mice (Figure 2 and data not shown). So in this model of transgenic mice, even if TRAIL overexpression does not induce apoptosis of the normal epithelial cells, it doesn't lead to an increase in proliferation of the cells either. It has previously been observed that in certain inflammatory conditions, such as rheumatoid arthritis, TRAIL displayed a bimodal action on the proliferation of synovial fibroblasts [28]. The chemical carcinogenesis protocol obviously induces inflammatory skin processes. The fact that despite an induction of inflammation we did not observe any significant effect of proliferation indicates that in the mouse *in vivo *inflammation *per se *does not result in a proliferative response towards excess TRAIL. It is possible that the effect observed in synoviocytes is cell type-specific or that, alternatively, it requires TRAIL doses, which were not reached in the transgenic animals.

The fact that none of the lesions formed following the DMBA/TPA treatment progressed into a squamous cell carcinoma (Figure 4A) was most probably due to the genetic background of the mice in which we performed the experiment. Indeed, mice from C57BL/6 background have previously been reported to be more resistant to tumor development and particularly to skin carcinogenesis, in contrast to mice having a DBA/2 genetic background which present a higher rate of tumors formation when subjected to the same protocol [41]. However, this resistance of benign tumors to progress to malignant lesions provided us with the opportunity to study the effect of high endogenous levels of TRAIL in skin on the early step of the skin tumorigenesis. Indeed, it is generally accepted from *in vitro *experiments and results obtained with xenografts *in vivo *that only fully transformed cells are sensitive towards the apoptogenic action of the TRAIL signaling pathways. This selectivity has been functionally linked to the expression of oncogenes such as Ras and Myc [42-46] but no coherent general mechanism has evolved that would account for the observed tumor-selectivity in a variety of experimental systems [discussed by [25]]. In some studies an effect of TRAIL on premalignant cells has been observed, but this effect depended on the combination with other agents, such as all-*trans*-retinyl acetate. Most notably, all-*trans*-retinyl acetate sensitized premalignant adenoma cells of APC-deficient mice to TRAIL-induced apoptosis, thus establishing a paradigm for chemoprevention of colon cancer by retinoic acid-TRAIL combination therapy [47].

In our model however, increased endogenous levels of TRAIL alone were sufficient for pretransformed cells to induce a delay in the onset of the tumorigenesis and a statistically significant decrease in the number of these lesions in TRAIL transgenic animals, if compared to wild-type littermates (Figure 4C). It will be interesting to test the effect of other drugs, such as all-*trans*-retinyl acetate or even standard chemotherapy for synergistic effects with high TRAIL levels. If confirmed using other models of transgenic mice and other models of tumorigenesis, this observation that TRAIL expression in a particular cell type can play a protective role at the earliest stage of tumorigenesis is of great interest. Indeed, the present model can be readily adapted to study the effects of high levels of endogenous TRAIL in a particular organ alone or in combination with TRAIL "sensitizers" [48-50] or other cancer therapeutics. Given the ongoing efforts to develop increasingly improved mouse models mimicking particular human tumors, such as the widely used MMTV-erbB2 transgenics for breast cancer, the present mouse model constitutes a versatile preclinical model to assess possible synergies between TRAIL-based therapeutics, such as recombinant TRAIL derivatives, TRAIL mimics or agonistic TRAIL receptor antibodies, and other cancer therapeutics in a organ and tumor type-specific manner.

## List of abbreviations

TRAIL: tumor necrosis factor-related apoptosis-inducing ligand; FADD: FAS-associated protein with death domain; DISC: death-inducing signaling complex; DMBA: 7,12-dimethyl-benz-anthracene; TPA: 12-*0*-tetradecanoylphorbol-13-acetate; SCC: squamous cell carcinoma.

## Conflict of interests

The authors declare that they have no competing interests.

## Authors' contributions

VK participated to the conception of the study, carried out the experimental design, performed the experiments and drafted the manuscript. SM analyzed the histological sections and performed the classification of the lesions. She read and approved the manuscript. HG conceived the study and participated in its design, and helped to draft the manuscript. All authors read and approved the final manuscript.
